# Digital hand‐held arthrometry is a reliable and accurate adjunct for diagnosing acute anterior cruciate ligament tears

**DOI:** 10.1002/jeo2.70251

**Published:** 2025-04-28

**Authors:** Richard Norris, Alan Price, Thomas W. Maddox, William Boswell, Cronan Kerin, Rachel A. Oldershaw

**Affiliations:** ^1^ Department of Trauma and Orthopaedics, Aintree University Hospital Liverpool University Hospitals NHS Foundation Trust Liverpool UK; ^2^ Department of Musculoskeletal and Ageing Sciences, Institute of Life Course and Medical Sciences, Faculty of Health and Life Sciences University of Liverpool Liverpool UK; ^3^ Therapies Department Aintree University Hospital, Liverpool University Hospitals NHS Foundation Trust Liverpool UK; ^4^ Small Animal Teaching Hospital, Institute of Infection, Veterinary and Ecological Sciences, Faculty of Health and Life Sciences University of Liverpool Wirral UK; ^5^ Radiology Department Aintree University Hospital, Liverpool University Hospitals NHS Foundation Trust Liverpool UK; ^6^ MRC‐Versus Arthritis Centre for Integrated research into Musculoskeletal Ageing (CIMA), Institute of Life Course and Medical Sciences, Faculty of Health and Life Sciences University of Liverpool Liverpool UK

**Keywords:** acute knee injury, anterior cruciate ligament, intrarater reliability, Lachman test, Lachmeter®, predictive validity

## Abstract

**Purpose:**

To evaluate the intrarater reliability and predictive validity of Lachmeter® measurements for diagnosing acute anterior cruciate ligament (ACL) tears, and to propose diagnostic thresholds.

**Methods:**

Lachmeter® measurements were recorded during the stabilised Lachman test for consecutive participants presenting to an acute knee injury clinic within 21‐days of injury. Intrarater reliability for individual limb and side‐to‐side (STS) difference (injured limb minus uninjured limb) measurements was investigated using a cross‐sectional, repeated‐measures design and the intraclass correlation coefficient (ICC). The predictive validity of STS difference and injured limb measurements was investigated using a prospective cohort design; sensitivity, specificity, negative (LR−) and positive likelihood ratios (LR+) were calculated using magnetic resonance imaging as the reference standard.

**Results:**

Intrarater reliability was excellent for individual limb and STS difference measurements in 102 participants. Of the 63 participants included in the validity analysis, 31 had a normal ACL and 32 had an ACL tear. LR‐ point estimates for STS differences <1.4 mm (0.07 [95% confidence interval [CI]: 0.02–0.29]) or injured limb measurements <7.5 mm (0.09 [95% CI: 0.02–0.34] produced ‘large’ shifts in the probability of ruling out an ACL tear. LR+ point estimates for STS differences ≥3.8 mm (10.67 [95% CI: 2.68–42.51]) or injured limb measurements ≥11.8 mm (10.67 [95% CI: 1.42–80.26]) produced ‘large’ shifts in the probability of ruling in a full‐thickness ACL tear.

**Conclusion:**

In participants presenting within 21‐days of knee injury, intrarater reliability was excellent for Lachmeter® measurements recorded during the stabilised Lachman test. Based on predictive validity estimates, Lachmeter® measurements can be used to differentiate normal from torn ACLs in acute presentations, but not partial from full‐thickness ACL tears. Diagnostic thresholds are proposed based on STS difference and injured limb measurements, and with consideration of the Lachman end point.

**Level of Evidence:** Level I.

**Abbreviations**
ACL: anterior cruciate ligament
ACLs: anterior cruciate ligaments
AED: accident and emergency department
AKIC: acute knee injury clinic
AUC: area under the curve
CHAMP: CHecklist for statistical Assessment of Medical Papers
CI: confidence interval
COSMIN: Consensus‐based Standards for the selection of health Measurement Instruments
FT: full thickness
GRRAS: Guidelines for Reporting Reliability and Agreement Studies
ICC: intraclass correlation coefficient
LoA: limits of agreement
LR+: positive likelihood ratio
LR‐: negative likelihood ratio
MDC: minimal detectable change
mm: millimetres
MRI: magnetic resonance imaging
NHS: National Health Service
OLS: ordinary least squares
PT: partial thickness
Q‐Q plots: quantile quantile plots
QUADAS: Quality Assessment of Diagnostic Accuracy Studies
ROC: receiver operator characteristic
SD: standard deviation
SEM: standard error of measurement
Sn: sensitivity
Sp: specificity
SPSS: statistical product and service solutions
STARD: Standards for Reporting Diagnostic accuracy studies
STS: side‐to‐side

## INTRODUCTION

Anterior cruciate ligament (ACL) tears are common, accounting for approximately half of all knee injuries presenting to an accident and emergency department (AED) with a traumatic haemarthrosis. Early detection of an ACL tear is critical to initiate treatment and mitigate the risk of further injury [11], but clinical examination of an acutely injured knee can be difficult, with less than 30% of ACL ruptures being correctly identified on initial assessment [1]. Magnetic resonance imaging (MRI) has been shown to be more accurate than clinical examination in acute presentations [13] but MRI assessment is contraindicated for certain individuals, expensive, and prolonged waiting times to acquire the images and report the results can further delay the diagnosis.

Historically, the Lachman and pivot shift have been considered the most accurate clinical tests for assessing the ACL following acute knee injury [4]. Based on the most recent systematic review with meta‐analysis in acute presentations, the pivot shift and Lever sign tests demonstrate the highest diagnostic accuracy for ruling in and ruling out an acute ACL tear respectively [34]. Positive likelihood ratio (LR+) point estimates for the pivot shift produce a ‘large’ shift in the probability of ruling in an acute ACL tear, but this test also demonstrates the highest false negative rate [34]. Negative likelihood ratio (LR−) point estimates for the Lever sign test are comparable with the Lachman and anterior drawer tests, producing only ‘small’ shifts in the probability of ruling out an acute ACL tear [34], highlighting the limitations of individual clinical tests.

Lachman testing requires the examiner to subjectively evaluate the amount of anterior tibial translation and the ACL end point [21]. The Rolimeter® (Aircast, Germany) is a hand‐held arthrometer that quantifies anterior tibial translation and has been shown to provide reliable measurements following acute knee injury [10]. This device has been validated against gold‐standard instrumented arthrometry [2, 31], with side‐to‐side (STS) differences in anterior tibial translation ≥3 mm considered diagnostic of an ACL tear [16]. However, the Rolimeter® has an analogue gauge that measures in 2 mm increments, making it difficult to evaluate STS differences with precision [15]. The Lachmeter® (Sao Paulo, Brazil) is a modification of the Rolimeter®, with a digital gauge measuring in 0.1 mm increments [27]. The Lachmeter® demonstrates greater precision than the Rolimeter® [20], with moderate‐to‐excellent reliability in post‐acute presentations [27], but its reliability and predictive validity have not been investigated following acute knee injury.

Measuring anterior tibial translation with greater precision could facilitate early identification of an ACL injury, therefore the primary objective of this study is to investigate the intrarater reliability and predictive validity of Lachmeter® measurements for diagnosing ACL tears within 21‐days of knee injury. Index testing will be conducted by one extended scope physiotherapist, blinded to all clinical information, before reference testing is performed. The first hypothesis is that validity point estimates will be superior to pooled estimates reported in the most recent meta‐analysis of clinical tests for acute ACL tears (Sn: 0.79, Sp: 0.91, LR+: 7.63, LR−: 0.23) [34]. The second hypothesis is that there will be a significant difference in STS measurements between normal, partial, and full‐thickness ACL tears. The secondary objective is to determine diagnostic thresholds for STS differences and absolute measurements of the injured limb, with and without consideration of the Lachman end point.

## METHODS

### Study design

Intrarater reliability was investigated through a cross‐sectional, repeated‐measures design, while validity was investigated using a prospective cohort design. To mitigate bias related to the study design, signalling questions from the Consensus‐based Standards for the selection of health Measurement Instruments (COSMIN) [23] and Quality Assessment of Diagnostic Accuracy Studies (QUADAS‐2) [37] risk of bias tools were considered during the design phase. The study is reported using the Guidelines for Reporting Reliability and Agreement Studies (GRRAS) [19], STARD 2015 guidelines [6], and the CHecklist for statistical Assessment of Medical Papers (CHAMP) [22].

### Sample size

For assessment of validity, with an anticipated sensitivity and specificity of 0.91 [15] and prevalence of 50% of the target condition [26], a minimum sample size of 56 was required to determine the sensitivity and specificity with a precision of 0.15 and 95% confidence. To account for a potential 10% attrition rate, the sample size was increased to 62. To meet the COSMIN recommendations for an ‘excellent’ reliability sample size, a sample of ≥100 is recommended [36]. Since all participants would be eligible for the reliability arm of the study, but participants would only be eligible for the validity arm if they underwent an MRI scan, data were collected until the minimum sample sizes for reliability (*n* = 100) and validity (*n* = 56) were reached.

### Study population

The study population was sampled from patients attending the outpatients Acute Knee Injury Clinic (AKIC) at Liverpool University Hospitals NHS Foundation Trust. Patients are referred to the AKIC if they present to an AED with a traumatic knee injury but no clinical or radiographic evidence of fracture. Testing was performed in the clinical setting and patients were managed according to their individual presentations. The decision to refer participants for MRI examination was based the Trust's clinical pathway (e.g., suspected extensor mechanism injury, joint instability and extension deficit/locked knee/suspected traumatic meniscal tear).

### Inclusion/exclusion criteria

Consecutive patients presenting to the AKIC within 21‐days of knee injury were approached by direct invitation and deemed eligible for inclusion if they were aged 18‐years or older and willing and able to give informed written consent. This time frame was selected as it is the most frequently reported threshold to differentiate acute (<3 weeks) from post‐acute (>3 weeks) knee injuries [33]. Patients were excluded from the study if they had skin conditions that precluded testing and excluded from the validity study if they had a previous history of ipsilateral or contralateral ACL injury (known ACL deficiency or reconstruction). Excluding patients with previous ipsilateral ACL injury was deemed necessary to ensure Lachmeter® measurements were reflective of the acute injury, while a history of contralateral ACL injury could skew STS difference measurements as the reference limb would not be ‘normal’. Patients were also excluded if they had contraindications to MRI examination or failed to attend their MRI scan appointment. Index testing was conducted in one outpatient orthopaedic department before commencing further assessment.

### Reference standard

MRI examination (1.5 T or 3.0 T Philips Ingenia MR system) was used as the reference standard as it demonstrates high criterion validity for ACL injury [28, 32] and the routine use of arthroscopy (gold standard) is not appropriate following traumatic knee injury [30]. The ACL status was classified as normal, partial thickness or full‐thickness tear based on the primary and secondary signs of injury [5]. All MRI scans were reviewed by one consultant musculoskeletal radiologist with 13 years' experience as a consultant.

### Timing and flow

Index testing was performed before any other subjective or objective information was collected. To ensure the ACL status had not changed between index and reference testing, participants reporting additional injury between the index and reference tests were excluded from the validity study. The same reference standard was used for all participants and all participant data were included in the analyses.

### Assessors

**Figure 1:**
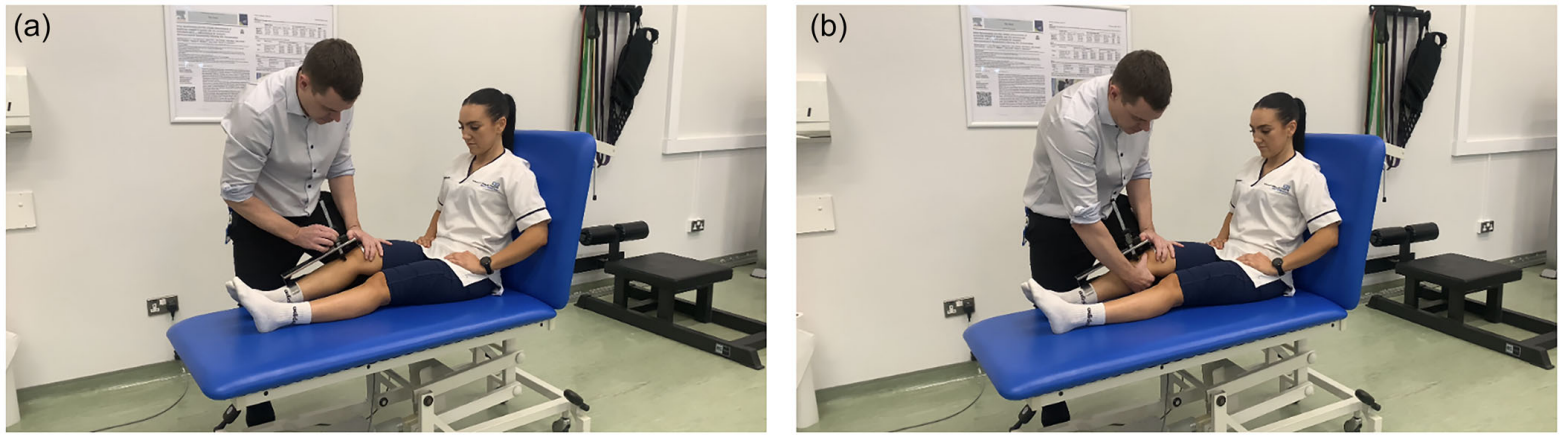
Representative images of the testing procedure. (a) Placement of the Lachmeter® and zeroing of the gauge. (b) Performing the stabilised Lachman test with the Lachmeter® in situ.

The assessor was a physiotherapist with 21‐years' clinical experience and 19‐years' experience working in an AKIC. This assessor had 12 years' experience using a Rolimeter® and 1‐years' experience using a Lachmeter®. Procedure: Supporting Information: Video S3 and Figure 1.

On entering the examination room, potential participants were asked to confirm their age and date of injury. Potential participants aged 18‐years or older, reporting an injury within the preceding 21‐days, were asked to lie on a cushioned examination bed (Huntleigh AKRON, Ipswich, UK) with the backrest upright. The assessor placed their knee under the participant's distal thigh so that the knee to be tested was in approximately 20°–30° of flexion; performing a Lachman test in this position has been previously described as the stabilised Lachman test [38]. The proximal support of the Lachmeter® was placed on the centre of the patella and the stem adjusted until it was in line with the tibial tuberosity. With the participant as relaxed as possible, the stem was pushed down gently until it contacted the tibial tuberosity. The digital display was zeroed, then the assessor performed a Lachman test with the Lachmeter® in situ. The assessor was blinded to the digital display during testing.

On completion of each trial, the Lachmeter® was removed from the leg and the measurement recorded to one decimal place. To reduce the impact of random error (equipment and/or assessor) and exclude unusual data points, the testing procedure was repeated on each leg until there were three measurements with ≤10% difference. This was deemed appropriate to ensure the assessor was confident with their measurements and test outcome, which reflects clinical practice. The mean of these three measurements was used for analysis, as recommended by the manufacturer; mean values also provide a more reliable measurement [7]. The Lachman end point was recorded as ‘hard’ (suggesting the ACL is normal) or ‘soft’ (suggesting the ACL is torn); if the end point was ambiguous or limited by participant guarding, it was recorded as ‘equivocal’. After both legs were tested, the procedure was repeated to determine intrarater reliability. All data were stored on a password‐protected Microsoft Excel spreadsheet.

### Randomisation

The order of testing for limbs (injured or uninjured) was randomised to control for potential sources of systematic error, including participant guarding or relaxation from repeated testing, using an online random item generator (www.random.org/lists).

### Blinding

The assessor performed the index test without knowledge of any clinical information and was not allowed to talk to the participants until testing was complete, other than to explain the nature of the study, that testing was standard clinical practice, and to provide instructions/information during testing. Lachmeter® testing was not performed on any participant prior to index testing. The assessor was blinded to the measurement until each individual trial was complete but was not blinded to the previous trial measurements. Participants were blinded to their results until all testing was complete. All MRI scans were performed after index testing and reported without knowledge of the index test results. MRI reports were cross referenced with the index test outcomes by an independent assessor.

### Data analysis

All statistical analyses were performed using SPSS 29.0 (SPSS Inc, Chicago, Illinois, USA). Continuous variables were assessed for normality by graphical (histograms and normal Q‐Q plots) and numerical analyses (Kolmogorov–Smirnov and Shapiro–Wilk tests). All relevant assumptions for the statistical tests were considered, and point estimates are reported with 95% confidence intervals (CI) where appropriate.

Descriptive statistics are presented for participant demographic variables and the time elapsed between assessments. Mean or median Lachmeter® measurements are presented for STS differences (injured limb value minus uninjured limb value) and individual limbs. Parametric or non‐parametric tests were performed, as appropriate, to determine whether there was a significant difference in measurements between participants with normal ACLs, partial ACL tears or full‐thickness ACL tears.

Intrarater reliability was assessed through the intraclass correlation coefficient (ICC) using a two‐way mixed effects model for absolute agreement based on the mean of three measurements (ICC_3,3_) [18]. The ICCs were categorised as ‘poor’ if less than 0.50, ‘moderate’ between 0.50 and 0.75, ‘good’ between 0.75 and 0.90, and ‘excellent’ if greater than 0.90 [18]. Measurement error was expressed as the standard error of measurement (SEM) [35] and calculated as SD×√(1‐ICC), where SD is the standard deviation of the differences between test and retest scores. The minimal detectable change (MDC), which reflects the smallest within‐person change that can be interpreted as ‘real’, above measurement error, was calculated as 1.96 ×√2 ×SEM [35].

Bland–Altman plots were constructed, with limits of agreement (LoA) reported, to assess systematic bias between test and retest measurements for the injured limb, uninjured limb, and STS differences. Evidence for fixed or proportional bias was assessed via ordinary least squares (OLS) regression [17], with the null hypothesis that the slope of the regression line equals zero, and the 95% CIs for the Y intercept and slope coefficient would include zero.

Receiver operator characteristic (ROC) curves were constructed and the area under the curve (AUC) calculated to determine how well the classification model distinguished between participants with normal ACLs, partial tears, or full‐thickness tears. The AUC was categorised as ‘no discrimination’ if 0.5, ‘poor’ between 0.5 and 0.7, ‘fair’ between 0.7 and 0.8, good between 0.8 and 0.9, and ‘excellent’ between 0.9 and 1.0 [25]. The ROC curve optimal cut‐off point, which represents the value with the highest true positive and lowest false positive rate, was identified using Youden's index both for STS differences and injured limb measurements.

Contingency tables (number of true positives, false positives, true negatives, and false negatives) were constructed by cross tabulating the initial index test results and MRI outcomes. Index test outcomes were categorised based on the manufacturer's guidelines (STS difference 0–3 mm = no ACL injury: 3–4 mm = partial ACL injury: >4 mm = complete ACL injury) and ROC curve optimal cut‐off values. Validity estimates are also provided for each 0.1 mm increment, and with consideration of the Lachman end point. Sensitivity, specificity, and corresponding likelihood ratios were calculated from the contingency tables. For LR+, the shift in pre‐ to post‐test probability was categorised as ‘no change’ if 1, ‘minimal’ between 1 and 2, ‘small’ between 2 and 5, ‘moderate’ between 5 and 10, and ‘large’ if greater than 10. For LR‐, the shift was categorised as ‘no change’ if 1, ‘minimal’ between 1 and 0.5, ‘small’ between 0.5 and 0.2, ‘moderate’ between 0.2 and 0.1, and ‘large’ if less than 0.1.

## RESULTS

**Figure 2:**
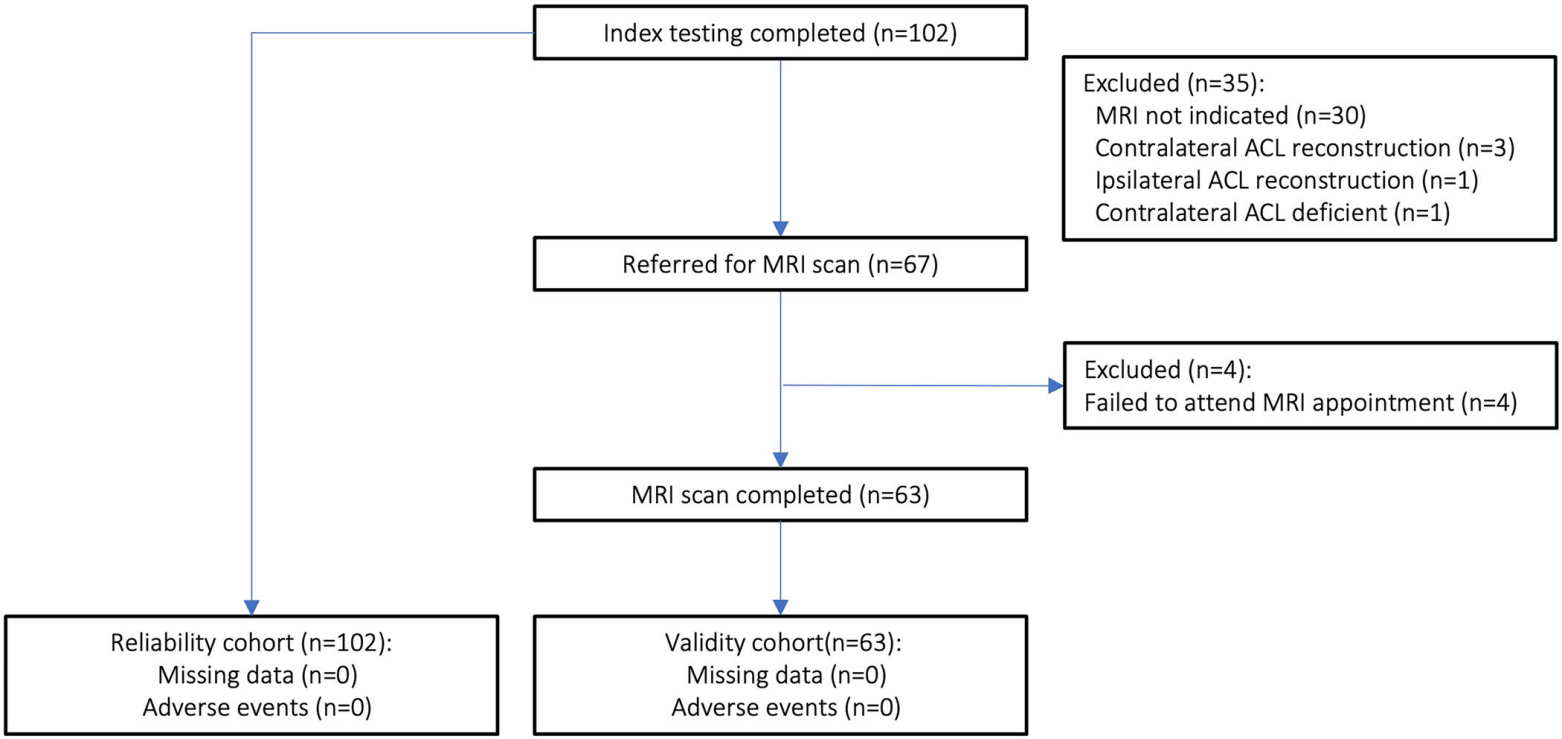
Flow of eligible participants from recruitment to the index testing session. ACL, anterior cruciate ligament; MRI, magnetic resonance imaging.

A total of 102 participants were recruited, nine (8.8%) of whom were excluded from the validity study due to a previous history of ACL injury or failure to attend their MRI scan appointment (Figure 2). All 102 participants (100%) were included in the reliability analyses; 63 (61.8%) underwent MRI examination and were included in the validity analyses. Of these 63 participants, 31 (49.2%) had a normal ACL, five (7.9%) had a partial ACL tear, and 27 (42.9%) had a full‐thickness ACL tear. Only two (7.4%) of the full‐thickness ACL tears were reported without concomitant injury (Supporting Information: MRI Data S2). No adverse events occurred during index or reference testing (0%) and no data were missing (0%). There was no significant difference in age, time from injury to index test, or time from index test to MRI examination between groups (Table 1).

**Table 1:** Participant demographics, time from injury to index test and index test to MRI, and Lachmeter® measurements for side‐to‐side (STS) difference and injured limb measurements.

			Validity cohort (*n* = 63)	
	Reliability cohort (*n* = 102)	Normal ACL (*n* = 31)	Partial ACL tear (*n* = 5)	FT ACL tear (*n* = 27)
Age: years	28.0 (16.5)	31.0 (14.5)	38.0 (13.0)	24.0 (11.0)
Gender: male (%)	65 (63.7)	20 (77.4)	4 (80.0)	21 (77.8)
Laterality: left (%)	58 (56.9)	19 (61.3)	4 (80.0)	6 (22.2)
Injury to index test: days	10.1 ± 4.1	9.7 ± 4.8	11.4 ± 3.8	10.0 ± 4.0
Index test to MRI: days	21.0 (20.5)^a^	18.0 (20.0)	33.6 ± 15.4	26.4 ± 16.5
STS difference: mm	1.6 (4.1)	0.2 (1.0)	3.7 ± 1.4	4.1 ± 2.2
Injured limb: mm	6.1 (2.0)	6.5 (3.0)	10.1 ± 2.3	10.8 ± 2.0

*Note*: Values are presented as mean ± standard deviation for normally distributed data and median (interquartile range) for non‐normally distributed data.

Abbreviations: ACL, anterior cruciate ligament; FT, full‐thickness; mm, millimetres; MRI, magnetic resonance imaging.

^a^ Days from index test to MRI for participants in the reliability cohort that underwent MRI (n = 63).

### Reliability cohort

Intrarater reliability was ‘excellent’ for individual limb measurements and STS differences (Table 2). The SEM was lower for the injured limb than the uninjured limb. The 95% CIs for MDC indicate that, with 95% confidence, STS differences of up to 0.5 mm of could be attributed to measurement error.

**Table 2:** intrarater reliability of Lachmeter® measurements.

Measurements (*n* = 102)	ICC [95% CI]	SD (mm)	SEM (mm) [95% CI]	MDC (mm) [95% CI]
Injured limb	0.985 [0.975–0.991]	0.60	0.07 [0.06–0.09]	0.21 [0.17–0.25]
Uninjured limb	0.965 [0.948–0.976]	0.66	0.12 [0.10–0.15]	0.34 [0.28–0.41]
STS difference	0.971 [0.956–0.980]	0.89	0.15 [0.12–0.18]	0.42 [0.34–0.50]

Abbreviations: CI, confidence interval; ICC, intraclass correlation coefficient; MDC, minimal detectable change; mm, millimetres; SD, standard deviation; SEM, standard error of measurement; STS, side‐to‐side.

**Figure 3:**
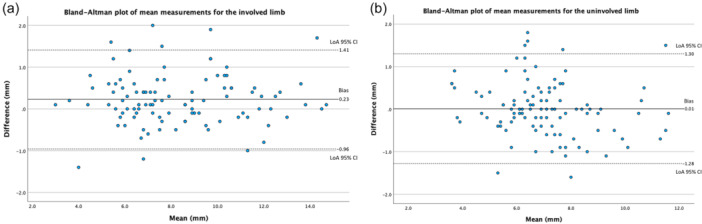
Bland–Altman plots with bias and limits of agreement (LoA) for the injured limb (a) and uninjured limb (b).

Bland–Altman bias was 0.23 mm (LoA: −0.96 to 1.41) for injured limb measurements, 0.01 mm (LoA: −1.28 to 1.30) for uninjured limb measurements (Figure 3), and 0.22 mm (LoA: −1.53 to 1.97) for STS difference measurements. The 95% CIs for all Y axis intercepts and slope coefficients of the OLS regression lines contained zero, therefore no fixed or proportional bias was evident for STS differences or individual limb measurements.

### Validity cohort

Lachmeter® measurements were comparable between the partial and full‐thickness ACL groups, but lower for the normal ACL group (Table 1). There were 13 outliers in the normal ACL group, defined as data points that lie beyond the whiskers of the box plot; therefore, a Kruskal–Wallis H test was performed to determine whether median STS differences were significantly different between groups. Distributions were not similar between groups, as assessed by visual inspection of the box plots, and distributions were significantly different (*X* ^2^(2) = 58.255, *p* < 0.001). Pairwise comparisons were performed using Dunn's procedure with a Bonferroni correction for multiple comparisons [8]. Adjusted *p* values are presented; values are mean ranks unless otherwise stated. This post hoc analysis confirmed statistically significant differences in STS differences between the partial ACL tear (76.75) and normal ACL (31.71) groups (*p* = 0.001), and full thickness ACL tear (76.75) and normal ACL groups (*p* < 0.001), but not the partial and full‐thickness ACL tear groups (*p* = 0.999).

### Lachman end point

Of the 31 normal ACLs, 23 (74.2% [95% CI: 55.4%–88.1%]) end points were recorded as ‘hard’ with the remaining eight (25.8% [95% CI: 11.9%–44.6%]) recorded as ‘equivocal’. For the five partial ACL tears, four (80% [95% CI: 28.4%–99.5%]) end points were recorded as ‘soft’ and one as ‘equivocal’ (20% [95% CI: 0.5%–71.6%]). Of the 27 full‐thickness ACL tears, 18 (66.7% [95% CI: 46.0%–83.5%]) end points were recorded as ‘soft’ and nine (33.3% [95% CI: 16.5%–54.0%]) as ‘equivocal’.

### ROC curve analysis

**Figure 4:**
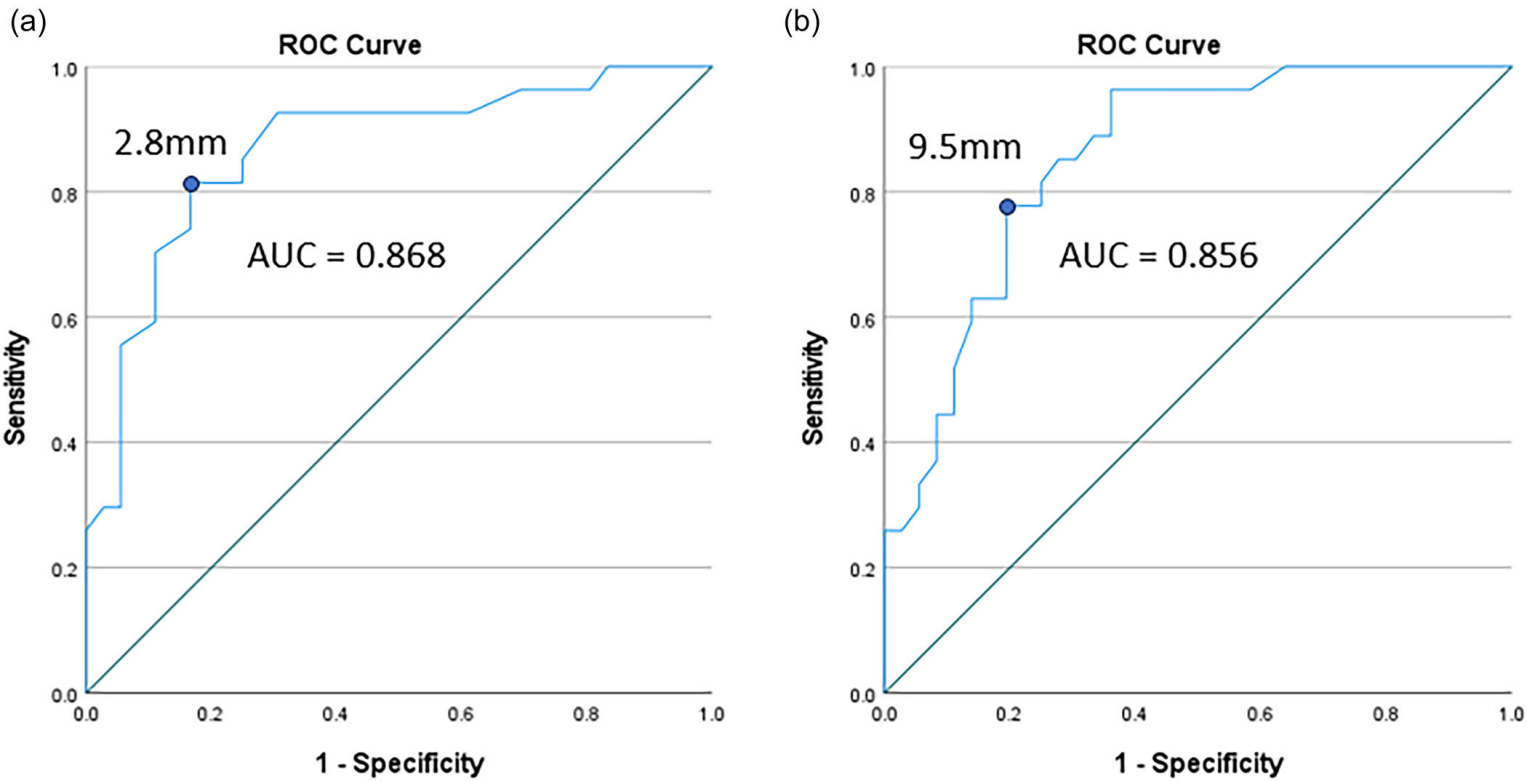
Receiver operator characteristic (ROC) curves, area under the curve (AUC), and optimal cut‐off points for full‐thickness ACL tears. (a) Side‐to‐side difference measurements, (b) absolute measurements of the injured limb.

There were three outliers for STS differences and absolute measurements of the injured limb. The optimal cut‐off values did not change with removal of the outliers; therefore, outliers were kept in the analyses. The AUC point estimates for STS differences were ‘fair’ for partial ACL tears (0.702 [95% CI: 0.565–0.838]) and ‘good’ for full‐thickness tears (0.868 [95% CI: 0.775–0.961]); the optimal cut‐off value was 1.9 mm for partial ACL tears and 2.8 mm for full‐thickness ACL tears (Figure 4). The AUC point estimates for injured limb measurements were ‘poor’ for partial ACL tears (0.666 [95% CI: 0.475–0.856]) and’ good’ for full‐thickness tears (0.856 [95% CI: 0.765–0.947]); the optimal cut‐off value was 9.2 mm for partial tears and 9.5 mm for full‐thickness tears (Figure 4).

### Predictive validity

Using the manufacturer's guideline discriminatory values, STS difference thresholds produced ‘small’ shifts in the probability of ruling out an ACL tear (<3 mm STS difference) and diagnosing a partial ACL tear (3–4 mm STS difference), but a ‘large’ shift in ruling in a full‐thickness ACL tear (>4 mm STS difference) (Table 3).

**Table 3:** Predictive validity estimates for Lachmeter® measurements based on the manufacturer's guidelines*, ROC curve optimal cut‐off values, and selected increments.

Diagnostic thresholds	ACL status	Sensitivity [95% CI]	Specificity [95% CI]	LR+ [95% CI]	LR− [95% CI]
<1.4 mm STS ∆	Normal	**0.94** [0.79–0.99]	**0.84** [0.66–0.95]	**5.81** [2.59–13.04]	**0.07** [0.02–0.29]
<3.0 mm STS ∆*	Normal	**0.75** [0.57–0.89]	**0.94** [0.79–0.99]	**11.63** [3.00–45.08]	**0.27** [0.15–0.49]
<7.5 mm injured limb	Normal	**0.94** [0.79–0.99]	**0.71** [0.52–0.86]	**3.23** [1.85–5.64]	**0.09** [0.02–0.34]
3‐4 mm STS ∆*	PT tear	**0.60** [0.15–0.95]	**0.88** [0.77–0.95]	**4.97** [1.83–13.48]	**0.45** [0.15–1.34]
≥2.8 mm STS ∆	FT or PT tear	**0.81** [0.63–0.92]	**0.94** [0.77–0.99]	**12.59** [3.26–48.62]	**0.20** [0.10–0.41]
≥9.5 mm injured limb	FT or PT tear	**0.72** [0.53–0.86]	**0.87** [0.70–0.96]	**5.57** [2.18– 14.26]	**0.32** [0.18–0.57]
≥2.8 mm STS ∆	FT tear	**0.81** [0.61–0.93]	**0.83** [0.67–0.93]	**4.89** [2.30–10.37]	**0.22** [0.10–0.50]
≥3.8 mm STS ∆	FT tear	**0.59** [0.39–0.78]	**0.94** [0.81–0.99]	**10.67** [2.68–42.51]	**0.43** [0.27–0.68]
≥4.0 mm STS ∆*	FT tear	**0.56** [0.35–0.75]	**0.94** [0.81–0.99]	**10.00** [2.49–40.09]	**0.47** [0.31–0.72]
≥9.5 mm injured limb	FT tear	**0.74** [0.54–0.89]	**0.81** [0.64–0.92]	**3.81** [1.89–7.68]	**0.32** [0.17–0.62]
≥11.8 mm injured limb	FT tear	**0.30** [0.14–0.50]	**0.97** [0.85–1.00]	**10.67** [1.42–80.26]	**0.72** [0.56–0.93]

Abbreviations: ACL, anterior cruciate ligament; CI, confidence intervals; LR−, negative likelihood ratio, LR+, positive likelihood ratio; mm, millimetres; STS ∆, side‐to‐side difference.

Using the ROC curve optimal cut‐off values (Table 3), STS differences <2.8 mm or injured limb measurements <9.5 mm produced ‘small’ shifts in the probability of ruling out an ACL tear. STS differences ≥2.8 mm produced a ‘large’ shift in the probability of ruling in an ACL tear (partial or full thickness), but only a ‘small’ shift in the probability of ruling in a full‐thickness tear. Injured limb measurements ≥9.5 mm produced a ‘moderate’ shift in the probability of ruling in an ACL tear (partial or full‐thickness), but only a ‘small’ shift in ruling in a full‐thickness tear. The likelihood ratios were superior when the Lachman end point was considered alongside the ROC curve optimal cut‐off values (Supporting Information: Data Spreadsheet S1).

**Figure 5:**
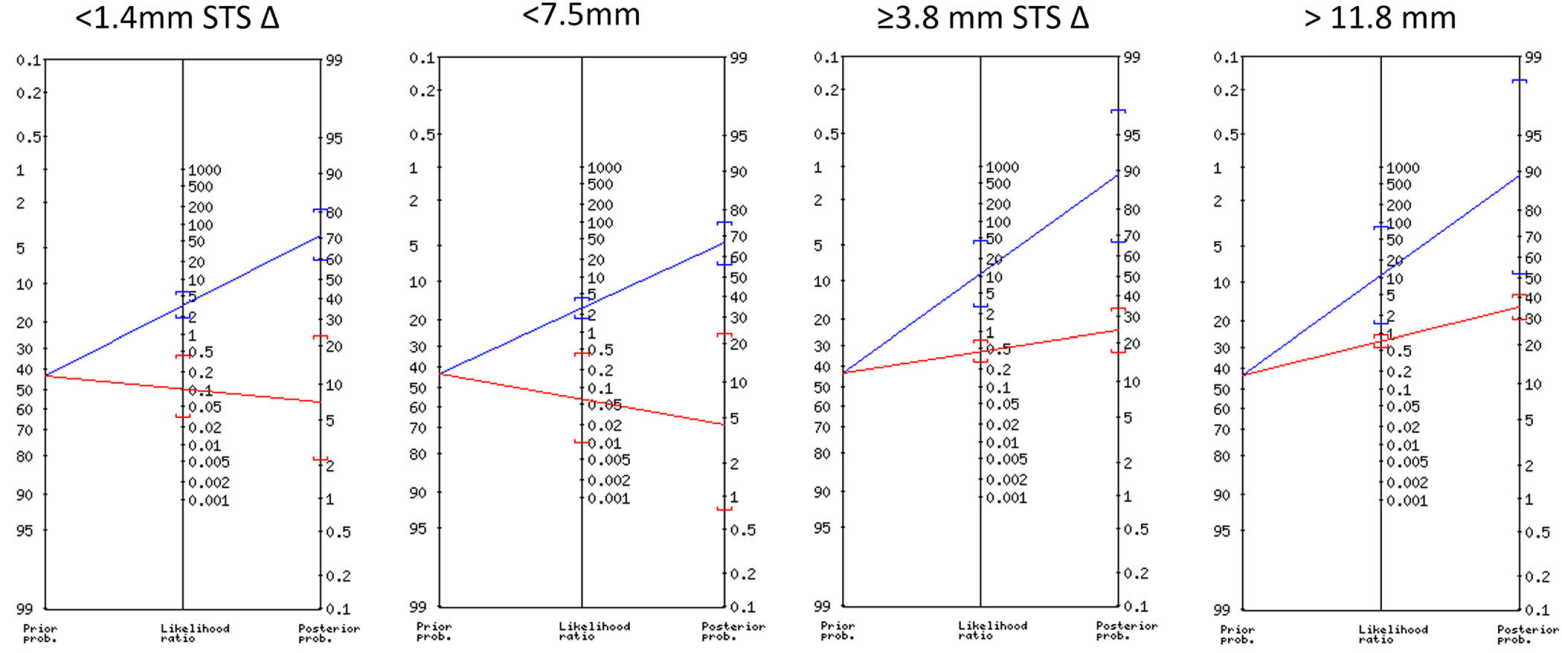
Fagan's nomograms illustrating the shift in the pre‐ to post‐test probability of diagnosing a full‐thickness anterior cruciate ligament tear based side‐to‐side difference (STS ∆) or injured limb Lachmeter® measurements. The pre‐test probability is 42.9% (prevalence of full‐thickness ACL tears in this study population), with the post‐test probability indicated by the blue (positive outcome) and red (negative outcome) lines. ACL, anterior cruciate ligament; STS ∆, side‐to‐side difference.

Based on 0.1 mm increments (Table 3, Figure 5 and Supporting Information: Data Spreadsheet S1), STS differences <1.4 mm or injured limb measurements <7.5 mm produced ‘large’ shifts in the probability of ruling out an ACL tear; when combined with the Lachman end point, the injured limb threshold increased to < 8.1 mm. Injured limb measurements ≥11.0 produced ‘large’ shifts in the probability of ruling in an ACL tear (partial or full thickness); when combined with the Lachman end point, this threshold decreased to ≥10.5 mm. STS differences ≥3.8 mm or injured limb measurements ≥11.8 mm produced ‘large’ shifts in the probability of ruling in a full‐thickness ACL tear; the Lachman end point did not affect these thresholds. Using ROC curve optimal cut‐off points and selected increments that produced ‘large’ shifts in the probability of ruling in or ruling out an ACL tear, thresholds for differentiating normal and torn ACLs using are proposed (Table 4).

**Table 4:** Thresholds for differentiating normal and torn ACLs using side‐to‐side differences or injured limb measurements.

ACL diagnosis	Side‐to‐side difference (mm)	Injured limb measurements (mm)
Normal	<1.4	<7.5
More likely normal	<2.8	<9.5
More likely partial or full‐thickness tear	≥2.8	≥9.5
Partial or full‐thickness tear	≥2.8	≥11.0
Full‐thickness tear	≥3.8	≥11.8

Abbreviation: ACL, anterior cruciate ligament.

Based on the Lachman end point alone, 18 (28.6%) tests could not be categorised as the end point was recorded as ‘equivocal’. If all ‘equivocal’ end points were categorised as positive, the sensitivity of the Lachman end point was 1.00 [95% CI: 0.89–1.00] and the specificity was 0.74 [95% CI: 0.55–0.88]; if they were categorised as negative, the sensitivity was 0.69 [95% CI: 0.50–0.84] and the specificity was 1.00 [95% CI: 0.89–1.00]. If the 2.8 mm STS difference threshold was applied to categorise ‘equivocal’ end points, the sensitivity was 0.91 [95% CI: 0.75–0.98] and the specificity was 0.97 [95% CI: 0.83–1.00], with the LR+ (28.09 [95% CI: 4.07–193.81]) and LR‐ (0.10 [95% CI 0.03–0.29]) point estimates producing ‘large’ shifts in the probability of ruling in and ruling out an ACL tear (partial or full thickness).

## DISCUSSION

The most important findings from the current study are that Lachmeter® measurements, recorded during the stabilised Lachman test, demonstrate excellent intrarater reliability in participants presenting within 21‐days of traumatic knee injury. Using specific thresholds, validity point estimates for ruling in and ruling out an ACL tear were superior to those reported in a recent meta‐analysis of clinical tests for acute ACL tears; [34] therefore, the first hypothesis was accepted. Although there was a significant difference in STS measurements between normal and torn ACLs, there was no significant difference between partial and full‐thickness ACL tears, therefore the second hypothesis was partially rejected. The optimal cut‐off points for full‐thickness ACL tears were 2.8 mm for STS differences and 9.5 mm for injured limb measurements. STS differences <1.4 mm or injured limb measurements <7.5 mm produced ‘large’ shifts in the probability of ruling out an ACL tear, while STS differences ≥3.8 mm or injured limb measurements ≥11.8 mm produced ‘large’ shifts in the probability of ruling in a full‐thickness ACL tear. Combining the Lachman end point result and Lachmeter® measurements produced ‘large’ shifts in the probability of ruling in and ruling out an ACL tear (partial or full thickness). Based on findings from this study, Lachmeter® measurements, recorded during the stabilised Lachman test, can be used to differentiate normal from torn ACLs in acute presentations, but not partial from full‐thickness ACL tears.

This is the first study to investigate the predictive validity of the Lachmeter® following acute knee injury and to combine hand‐held arthrometer measurements with the Lachman end point. This is also the first hand‐held arthrometer study to be reported in accordance with the GRRAS, STARD and CHAMP guidelines. Signalling questions from the COSMIN and QUADAS‐2 risk of bias tools were used during study design to mitigate bias, and the reliability sample size is considered ‘excellent’.

### Reliability

Reliability is a characteristic of a test used in a population, not just the test itself, and should be investigated in a sample in which the test is to be used [7]. ACL tears are caused by traumatic injury to the knee [9] and the intrarater reliability of Lachmeter® measurements was therefore investigated in a sample of patients presenting with an acute knee injury. Previous studies have investigated the reliability of hand‐held arthrometry in healthy participants or have employed case‐control study designs in post‐acute populations, which introduces selection bias [14, 16, 24, 27, 29]. Only one previous study has investigated the reliability of hand‐held arthrometry in participants with acute knee injuries [10], demonstrating a strong correlation between test and retest Rolimeter® measurements. Although these findings are consistent with reliability estimates in the current study, the devices used and timeframes between measurements were different. No previous studies investigating the reliability of the Rolimeter® or Lachmeter® have been reported using the GRRAS guidelines, which hinders assessment of risk of bias with these studies.

### Validity

Although STS differences in anterior tibial translation ≥3 mm are considered diagnostic of an ACL tear, only one previous study has investigated the predictive validity of this threshold using a hand‐held arthrometer [15]. In 34 participants presenting to a physiotherapy clinic within 10 days of knee injury, the sensitivity and specificity of Rolimeter® measurements were both reported as 0.91. However, it is not clear whether participants with partial ACL tears were included, or if the assessor was blinded to clinical information that could bias the test outcome. Furthermore, five participants (14.7%) had previous injuries to their contralateral knee, which could impact STS difference measurements.

The optimal cut‐off value for a full‐thickness ACL tear using STS differences identified in the current study was 2.8 mm, which is consistent with the recommended threshold of 3 mm. Although STS differences ≥2.8 mm did produce a ‘large’ shift in the probability of ruling in an ACL tear, the shift was only ‘small’ for ruling in a full‐thickness tear as partial ACL tear measurements were not significantly different from full‐thickness tear measurements. By applying the 2.8 mm STS difference threshold alone, 85.7% (54/63) of patients would have been correctly categorised as having a normal or torn ACL on initial consultation, which is considerably higher than 28.2% reported previously [1]. In participants with contralateral knee injuries, STS differences may be misleading, therefore absolute values for the injured limb were also investigated; mean and median injured limb measurements are consistent with mean values reported using the Rolimeter® [2, 10].

One outlier from the current study had a full‐thickness posterior cruciate ligament tear with a positive sag sign, producing a false positive injured limb measurement of 11.0 mm, and 5.1 mm STS difference. In this participant, the ACL end point was recorded as ‘hard’, highlighting the importance of combining the Lachman end point and Lachmeter® measurements. Although all ‘hard’ end points were recorded in participants with normal ACLs, and all ‘soft’ end points were recorded in torn ACLs, almost 30% of the end points were deemed ambiguous or limited by guarding from the participant. In this scenario, Lachmeter® measurements allow such ‘equivocal’ Lachman test outcomes to be categorised. If the end point was considered in combination with the 2.8 mm STS difference threshold for equivocal outcomes, the number of patients correctly diagnosed as having a normal or torn ACL at the initial consultation would have been 90.5% (57/63).

### Limitations and recommendations

All participants were referred from an AED so findings may not be applicable to patients presenting to non‐emergency departments. Index testing was performed within 21‐days of injury; therefore, results may not be generalisable to patients evaluated at later time points. All participants in the validity study had to be deemed appropriate for MRI, which may introduce selection bias, but routine MRI for all patients presenting to an AKIC is not indicated and represents an inefficient use of healthcare resources. Likewise, the routine use of arthroscopy following traumatic knee injury is not appropriate [30].

The Lachmeter® is a quick and simple adjunct to the stabilised Lachman test, which is lightweight, portable, easy‐to‐use, and accurate. However, unlike instrumented arthrometry, the force applied is not standardised, which is a potential source of measurement error between different assessors. In the current study, intrarater reliability was investigated within the same session, which was deemed appropriate as longer intervals (e.g., 1 week) may result in changes in measurements that could be attributed to the recovery process (e.g., fluctuations in pain, effusion and guarding) rather than measurement error. Within‐session testing also negated loss to follow up and ensured all data were collected within 21‐days of injury. Future studies should investigate the interrater reliability of Lachmeter® measurements following acute knee injury, and reliability studies should consider longer intervals before retesting. Until the interrater reliability of Lachmeter® measurements has been established, the current method of testing may be more appropriate for settings that rely heavily on the expertise of a single clinician, such as a specialist AKIC.

The assessor had significant experience examining patients following knee injury, including the use of the Rolimeter®, but only 1‐years' experience using the Lachmeter®, which requires a different handling technique. Although the assessor was blinded to the measurements during testing, the assessor was not blinded to the individual trial measurements, therefore it is possible that retest scores were influenced, and reliability estimates could be inflated. However, it was not practical to blind the assessor to each trial measurement by including an independent assessor ad hoc for the duration of the study. Testing was repeated until there were three Lachmeter® measurements within 10% of each other, for each individual leg, therefore the number of trials varied between some participants. This was to ensure unusual data points were excluded and the assessor was confident in their measurements, which reflects clinical practice. Future studies should investigate reliability in assessors with different levels of experience, with the assessor blinded to measurements where practical.

Of the 63 participants included in the validity analysis, 51.0% had an ACL tear, which is consistent with previous data reported for patients presenting to an AED following traumatic knee injury; [26] only five of these had partial tears. Interestingly, partial ACL tears demonstrated soft end points, with comparable STS difference and injured limb measurements to full‐thickness tears, highlighting the difficulty in differentiating between the two. Based on the Lachmeter® manufacturer's recommendations, the pivot shift test should be used to differentiate partial from full‐thickness tears when the injured limb measurements are 3–4 mm greater than the uninjured limb, but this was not investigated in the current study. The clinical relevance of the tear grade is contentious as up to one‐third of full‐thickness ACL tears may heal [12] and ACL reconstruction is indicated in those with symptomatic instability rather than tear grade [3]. Differentiating partial and full‐thickness ACL tears is unlikely to influence patient management. However, differentiating normal and torn ACLs is clinically relevant, and the current assessment method appears suitable for this purpose. Future studies with a larger number of partial ACL tears, and incorporation of the pivot shift test, are recommended.

Based on the findings from this study, the following clinical recommendations are proposed:

1. When the Lachman end point is unequivocally ‘soft’ or’ ‘hard’, use this to rule in/out an ACL tear.
2. When the Lachman end point is equivocal, use Lachmeter® measurements.
3. For patients with a normal contralateral knee, use STS difference thresholds.
4. For patients with a known contralateral ACL injury, use injured limb thresholds.
5. Consider the MDC for STS difference (0.4 mm) and injured limb measurements (0.2 mm).

## CONCLUSION

In participants assessed within 21‐days of traumatic knee injury, Lachmeter® measurements recorded during the stabilised Lachman test demonstrate excellent intrarater reliability. Based on predictive validity estimates, Lachmeter® measurements can be used to differentiate normal from torn ACLs, but not partial from full‐thickness ACL tears. When the Lachman end point is equivocal, Lachmeter® measurements can be used to improve the diagnostic accuracy of the test; diagnostic thresholds are proposed based on the findings.

## AUTHOR CONTRIBUTIONS

All authors contributed to the study conception and design. Material preparation, data collection and analysis were performed by Richard Norris, Alan Price, Thomas Maddox and Rachel Oldershaw. The first draft of the manuscript was written by Richard Norris and all authors commented on previous versions of the manuscript. All authors read and approved the final manuscript.

## CONFLICT OF INTEREST STATEMENT

The authors declare no conflict of interest.

## ETHICS STATEMENT

This study was approved by the NHS Health Research Authority, HRA and Health and Care Research Wales (22/NI/0147), and conducted in accordance with the ethical standards of the World Medical Association Declaration of Helsinki (2002). Informed consent was obtained from all individual participants included in the study. The authors affirm that the model provided informed consent for publication of the images in Figure 1A,B and Supplemental Video.

## Supporting information

Supporting information.

Supporting information.

Supporting information.

## Data Availability

The data sets used and/or analysed during the current study are available from the corresponding author on reasonable request.
